# Recycling of post-consumer plastic packaging waste in the EU: Recovery rates, material flows, and barriers

**DOI:** 10.1016/j.wasman.2021.04.002

**Published:** 2021-05-01

**Authors:** Ioannis Antonopoulos, Giorgia Faraca, Davide Tonini

**Affiliations:** European Commission Joint Research Centre, Calle Inca Garcilaso 3, 41092 Sevilla, Spain

**Keywords:** Purity, Circular economy, Sorting, Reprocessing, Material flow analysis, Recovery efficiency, DRS, deposit refund scheme, EC, European Commission, EU27, Europe with 27 member states, EPR, extended producer responsibility, EPS, expanded polystyrene, GHG, greenhouse gas, HDPE, high-density polyethylene, LDPE, low-density polyethylene, LLDPE, linear low-density polyethylene, LPW, light packaging waste, MFA, material flow analysis, MRF, material recovery facility, NIR, near-infrared spectroscopy, PE, polyethylene, PET, polyethylene terephthalate, PO, polyolefin, PP, polypropylene, PPW, plastic packaging waste, PRO, producer responsibility organisation, PS, polystyrene, PVC, polyvinylchloride, RDF, refuse derived fuel

## Abstract

Increasing plastic waste recycling is a milestone of European environmental policy to reduce environmental impacts and dependency on foreign resources. This is particularly challenging for plastic packaging waste, consisting of very heterogeneous fractions and typically rather contaminated. In this study, we collected primary data from plants sorting and recycling plastic packaging waste to illustrate process efficiencies, material flows, and barriers. We observed that significant losses of target materials occurred both at sorting and recycling stages. These were higher for polymers such as films, polypropylene and polystyrene, and lower for polyethylene terephthalate and high-density polyethylene. Applying material flow analysis, we estimated an overall end-of-life recycling rate for post-consumer plastic packaging waste in EU27 in 2017 of 14% (not considering waste exported as recycled; 25% otherwise). An improved scenario for 2030 showed that achieving an overall end-of-life recycling rate of about 49% was possible when best available practices and technologies were implemented. To fulfil the ambitious recycling targets set at EU27 level (55% overall recycling rate), substantial improvements are necessary at the plants, product design, collection system, and market level. Our findings further indicate that films and other problematic contaminants in the input-waste considerably hamper the recovery rates, thus the improvement of the efficiency of the collection systems is imperative. In parallel, the development of markets for lower value fractions, e.g. polypropylene, could be a way forward to increase recycling, while improvements in the product design will considerably reduce the presence of impurities and contaminants in the input-waste.

## Highlights

• We collected primary data on plants efficiencies and barriers hindering recycling.
• Recovery rates are higher for PET and HDPE and lower for films, PP, PS.
• Current EU27 PPW recycling rate is 15% excluding the exported waste (25% otherwise).
• Presence of films in the input-waste hampers recovery of target materials.
• Substantial improvements are needed in the recycling chain, primarily the collection.

## 1 Introduction

Recycling plastic packaging waste (PPW) is a milestone of the EU Circular Economy and Green Deal to reduce fossil fuel dependency, greenhouse gas (GHG) emissions, and impacts on biodiversity due to littering in uncontrolled disposal routes (European Commission, 2020). Striving to reach this goal, the parameters governing the quality, quantity and fate of post-consumer packaging prepared and sent for recycling are currently undergoing a period of reform, largely driven by policy changes at the EU level. The amendment to Directive 94/62/EC (European Parliament and the Council, 1994) on Packaging and Packaging Waste sets a recycling target of 50%, 55%, and 60% to be achieved by 2025, 2030, and 2035, respectively (European Parliament and the Council, 2018). In addition, new rules were introduced for the calculation of the achievement of these targets, highlighting the importance of improving the efficiency of the sorting and recycling facilities to maximise quality and minimise rejects, thus ensuring high-quality recycling. Within the post-consumer plastic waste fraction, 61% comes from packaging applications (PlasticsEurope, 2020). This said, in 2017 the amount of PPW separately collected for subsequent recycling amounted to only about 41% (EU27; Eurostat, 2019).

Material recovery facilities (MRFs, also referred to as sorting plants) and recycling plants are two key stages within the plastic mechanical recycling value chain. MRFs receive separately collected waste and further sorts it into specific streams (e.g. bales of polyethylene bottles). Recycling plants receive the sorted bales and reprocess them into secondary raw materials in the form of flakes/pellets/granules that can be readily used for consumers’ goods manufacture (e.g. packaging). Currently, the sorting and recycling rates vary greatly across the EU member states, depending on the quantity, type, purity and complexity of the materials received as well as on the number, type and sequence of technologies used at the plants (Villanueva and Eder, 2014, Cimpan et al., 2016, Ragaert et al., 2017). The decision on what polymers to target is based on the market demand and prices, technological setup but also on the composition and purity of the input materials. In particular, the presence of impurities may cause technical, economic and/or market barriers to the substitution of virgin plastics, as discussed in Faraca and Astrup, 2019, Rigamonti et al., 2018. The extended producer responsibility (EPR) systems usually establish minimum quality requirements for the input materials to ensure high quality recycling throughout the entire recycling process (Dri et al., 2018), although the design and the operation of the plants (e.g. availability of storage space, equipment installed, presence of additional manual sorting lines, or the designed throughput) also play an important role (WRAP, 2014). However, there are a number of factors influencing the quality of post-consumer packaging recycling, which often are not plants’ responsibility, such as the collection system in place and the product design, as illustrated in Eriksen et al. (2019). Here quality is intended as those recycled plastic properties (e.g. colour, odour, viscosity, heavy metal content, etc.), which can limit the uptake/functionality/secondary use of recyclates. Finally, the presence of impurities further influence costs, both as investment and revenues from selling the recyclates (Faraca et al., 2019).

Reviewing the literature, we observed that i) few studies exist with primary data on the recovery and purity rates of MRFs and recycling plants in EU and that ii) very few studies investigated operating plants about the concrete issues leading to decrease in output volume and quality. In relation to the first point, key studies identified were Cimpan et al., 2015, Mastellone et al., 2017, Brouwer et al., 2018, and Van Eygen et al. (2018). Cimpan et al. (2015), alongside reviewing the technical performances of MRFs, elaborates the recovery rates of a MRF in the UK using the sampling results obtained by Eule (2013) with tests under controlled conditions. Mastellone et al. (2017) investigates the material recovery rate of a MRF in Italy, under controlled testing conditions. Van Eygen et al. (2018) provides average data on MRFs in Austria, while Brouwer et al. (2018) for the Netherlands. Other studies use secondary data and/or best-guess estimates as input to life cycle assessments or material flow analyses (Pressley et al., 2015, Cimpan et al., 2016, Faraca et al., 2019), are at lab-scale (e.g. Thoden van Velzen et al., 2017) or refer to regions with very different collection and management practices (e.g. US; DEQ, 2011, Damgacioglu et al., 2020). While modelling of environmental impacts from PPW management have been flourishing in the last years (e.g. Andreasi Bassi et al., 2017, Choudhary et al., 2019), these mostly rely on outdated, geographically narrow and/or secondary data (e.g. Al-Salem et al., 2014, Hou et al., 2018, Rigamonti et al., 2014), despite the fact that models have been proved very sensitive to the use of such data (Bisinella et al., 2017, Faraca et al., 2019). To our knowledge, only Eule, 2013, Mastellone et al., 2017 performed specific investigations on individual MRFs, respectively in UK and Italy, to detect the issues leading to decrease in output volume and quality of recyclates. However, a detailed analysis of the barriers that plant operators face could reveal target actions that need to be implemented to achieve higher quality recycling.

To identify hotspots in a system and perform future forecast, modelling of how the different material streams interact throughout the value chain is often performed by material flow analysis (MFA). In general, MFA rely on the mass conservation principle for the system under assessment. This means that in the case of waste management the sum of inputs (waste generation + imports) equals the sum of outputs (production of recycled products + export + accumulation + losses). This type of analysis has proven robust and has been used to model plastic waste flows in a number of studies (e.g. Brouwer et al., 2018; Kawecki et al., 2018 for plastic waste and textiles in EU and Switzerland; Van Eygen et al., 2018 for PPW in Austria). While Brouwer et al., 2018, Van Eygen et al., 2018 analysed the implications of their results for a circular economy, these analyses cover the Dutch and the Austrian conditions only. A recent study by Tallentire and Steubing (2020) provides a general overview of packaging waste material flows in EU, whereas Eriksen et al. (2020) analyses the evolution of plastic flows over time under different system assumption, including quality-related aspects of the secondary plastic. However, both studies rely on secondary data, and do not focus specifically on the technical challenges of PPW management. Besides the identification of system hotspots by MFA, it is equally important to integrate such hotspots with an analysis of the barriers faced by plant operators to understand the actual technical challenges and suggest specific measures that can enhance plastic recycling and contribute to the achievement of future targets. We observe that this aspect is lacking in the available literature.

Against this background, the study contributes to advancing the knowledge in the field of post-consumer PPW recycling by fulfilling the following objectives: i) collecting primary data about PPW recovery and purity rates of MRFs and recycling plants operating in EU; ii) investigating on the issues leading to decreased volume and quality of the outputs by surveying the operators and visiting the individual plants; iii) broadening the perspectives by illustrating the MFA of the EU27-wide flows of PPW using the primary data complemented with the necessary literature. To achieve these aims, we selected five MRFs and eight recycling plants treating post-consumer PPW across Europe. We use the MFA as a means to pinpoint hotspots, discuss needs for improvements and barriers to fulfil the EU27 2030 recycling targets. This study contributes to better understand the drivers and barriers influencing the quantity and quality of the post-consumer PPW recycled in EU27. The figures elaborated through this study provide support to the plastic strategy of the European Commission (2018) and constitute a useful dataset for use in life cycle assessment of plastic waste management.

## 2 Materials and methods

**Fig. 1:**
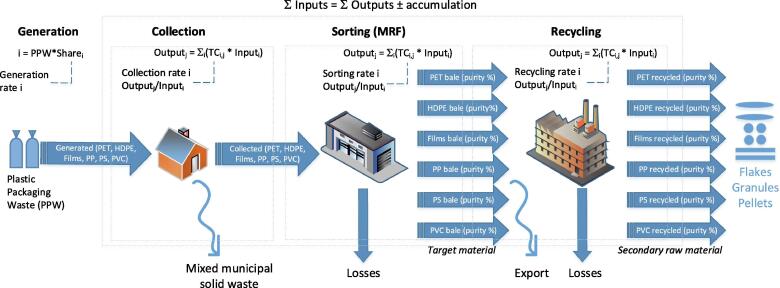
Conceptual diagram depicting the waste management phases considered in this study for plastic packaging waste. The dashed box represents the system boundaries for the material flow analysis (MFA). The dash-dot-dot boxes represent the individual waste management phases considered within the MFA. TC: transfer coefficient.

The waste management phases considered in this study are depicted in the conceptual diagram shown in Fig. 1. The next three sections explain: i) the terminology used in the study (Section 2.1), ii) the collection of primary data from 5 MRFs and 8 recycling plants across EU targeting PPW (2.2, 2.3) and ii) the MFA of post-consumer PPW for year 2017 and 2030 in EU27 (Section 2.4).

### 2.1 Definitions

The following definitions apply in this study:

*Capture rate (or separate collection rate)*: a quotient of mass between the amounts of a waste material segregated into a separate stream at the point of generation (including impurities) and the total mass generated of that material (%) (Directive 2008/98/EC) (Dri et al., 2018).

*Contaminants:* non-target material or chemicals that alter the physico-chemical properties of the secondary raw material (or recyclates).

*End-of-life recycling rate:* a quotient of mass between the output stream from a recycling plant (secondary raw material, e.g. recycled PET, including market-acceptable impurities) and the amount of *that* waste material generated after use (e.g. PET-waste generated), conforming to UNEP (2011).

*Films*: this category includes all flexible household plastic packaging, e.g. PE/LDPE films. Typically, it consists of bags, multilayer films and foils.

*Material recovery facility* (MRF): generic term for a specialized installation receiving different mixed waste streams containing recyclable materials and separating recyclable materials into different categories and material types (output products), using physical processing techniques, such as combinations of mechanical, pneumatic, sensor-based processes and some degree of manual sorting and quality control (Cimpan et al., 2015).

*Sorting rate*: a quotient of mass between the output stream from a MRF (i.e. the wet weight of the bale of the target recyclable, e.g. PET, including market-acceptable impurities) and the stream of the recyclable in input to the plant (Eq. (1)). While other definitions of sorting rate have been suggested (see Cimpan et al., 2015, Mastellone et al., 2017), we here apply Eq. (1) as it reflects parameters typically known by the plant operators and widely used in the sector.

(1) $$\frac{\mathrm{Bale}\;of\;target\;polymer\;incl.impurities(ww)}{\mathrm{Input}\;of\;polymer\;to\;the\;plant(ww)}\%$$

*Plastic packaging waste (PPW):* plastic waste generated from use of packaging (‘packaging’ definition in Directive 1994/62/ECb).

*Purity rate*: amount of target materials in the total amount of the separated or recovered (%) (based on Dri et al., 2018). The *impurity rate* equals 100%-purity.

*Rejects (or losses):* Material (both target and non-target*)* rejected from sorting processes and not included in process outputs destined to recycling.

*Recycling plant (or reprocessing plant)*: installation receiving (typically pre-sorted, e.g. by MRFs) streams of recyclable material and recycling them into a secondary raw material in the form of flakes/pellets/granules that can be readily used for production of consumer’s goods. It typically includes one or more sorting/treatment steps to further homogenise the material in input to the actual recycling technology.

*Recycling rate (or reprocessing rate)*: a quotient of mass between the output stream from a recycling plant (secondary raw material, e.g. recycled PET) and the total mass of the recyclable in input to the plant (e.g. PET bale, including market-acceptable impurities) (Eq. (2)). Also called recycling process-efficiency rate (UNEP 2011).

(2) $$\frac{\mathrm{Bale}\;of\;recycled\;target\;polymer\;incl.impurities(ww)}{\mathrm{Bale}\;of\;target\;polymer\;in\;input\;to\;the\;plant\;incl.impurities(ww)}\%$$

*Secondary raw material (recycled material or recyclate):* Material that has been sorted, reprocessed and prepared so that it is suitable for use directly in new product manufacture, without further sorting/preparation (e.g. clean, dry polymer flakes, pellets, or granules).

*Sorted fraction (or material):* A grade of material that has been sorted after collection but has not been sufficiently prepared to be a Secondary Raw Material.

*Target material*: The material or mix of materials that is the objective target by the sorting or recycling operation, e.g. PET bottles in a bale of PET.

### 2.2 Primary data collection from material recovery facilities and recycling plants

The surveyed plants, classified into MRF and recycling (REC) followed by a number, present differences in the source of input material received, target polymers and technological setup, as detailed in Table 1. The plants were located in Germany, France, Spain, Italy, Benelux, Scandinavia and Croatia, thereby covering different collection systems for implementing the Packaging Directive. The survey took place between June and November 2019. A detailed questionnaire was sent out to the surveyed plants, aiming at gathering two types of information: 1) process-related information, including questions concerning the collection method (e.g. commingled or single-stream, organised by the national EPRs or directly from deposit refund schemes, i.e. DRS), the mass and the composition of the flows in input and output as well as the purity of the output materials (detailed information in Supplementary Information, SI); and, 2) quality-related information, investigating current barriers and opportunities faced by plant operators to improve the efficiency and purity of the secondary raw material.

**Table 1:** Overview of the surveyed plants. LPW: light packaging waste, DRS: Deposit Refund Scheme; the rest abbreviations may be found in the dedicated section.

**Plant ID**	Collection system*	Target polymer*	**Technological setup**
MRF1	LPW	PET, PP, PS, film, HDPE	Manual pre-sorting, sieve, ballistic separator (x2), NIR (x6), magnet, manual sorting, baler
MRF2	LPW	PET, HDPE, films	Manual pre-sorting, trommel, ballistic separator, vacuum (x3), film baler, magnet (x2), NIR (x5), manual sorting, Eddy current separator, baler
MRF3	LPW	PET, PP, films, PS, HDPE	Bag opener, trommel, ballistic separator, magnet (x2), vacuum, NIR(x5), HD camera, vibrating screener, Eddy current separator, baler
MRF4	LPW	PET, PS, HDPE, LDPE	Feed hoper, bag opener, drum screen, magnet (x3), air classifier, rotary feeder, NIR(x4), Eddy current separator, Manual sorting, baler
MRF5	LPW	PET, HDPE, films	Reception of inputs, bag opener, ballistic separator, magnet (x2), manual sorting (x2), Eddy current separator, baler
REC1	Household plastic films from MRF	PP, HDPE	Shredder, magnet, cascade screen NIR (x2), shredder, friction washer, dryer, extruder
REC2	PET from MRF	PET	Manual bale breaker, metering unit/hopper, manual sorting, flakes, baler
REC3	PET from MRF	PET	Bale opener, magnet (x2), Eddy current separator (x2), NIR (x2), sink/float separation, centrifuge, hot wash, centrifugal dryer, pelletizing
REC4	PET from DRS and MRF	PET, PP, HDPE	Bale opener, magnet, label remover, ballistic separator, Eddy current separator, NIR (x3), manual sorting, hot washing, sink/float separation, air classification, extruder (x2), flake sorter, baler
REC5	PET bottles from DRS & household mixed LPW from DRS	PET	Bale opener/hopper, manual sorting, grinding, hot wash and centrifuge (x2), density separator (x2), NIR (x3), bagging
REC6	Sorted bales of PP & HDPE from MRF	HDPE, PP	Hopper/shredder, magnet, air classifier, drum screen washing, density separator, extrusion, bagging
REC7	Mixed plastics packaging from bring banks & PET bottles from DRS	PET, HDPE, PP, film	Feed hopper, film splitter, trommel, air classifier, NIR (x5), wind sifter, manual sorting, baler, granulation, density separation (x2), turbo wash, centrifuge dryer, air filter, bagging
REC8	LPW from separate collections	HDPE, PP, PS	Feed hopper, trommel, air classifier, magnet, NIR (x3), baler, hot wash and centrifuge, density separator (x2), extruder, bagging

* Detailed information about the inputs and source of inputs are presented in the SI document.

The surveyed plants targeted PET, HDPE, PP, PS and films (LDPE/PE). Films were considered in this study as made of LDPE, but were singled out as a type of plastic packaging product because their physico-chemical properties require specific sorting and recycling technologies (Horodytska et al., 2018). Process-related information on sorting and recycling of colour-separated PET, HDPE and film was aggregated into one fraction (e.g. PET), since very few operators out of the surveyed plants target colour-separated polymers. In contrast, the quality-related information about colour-separated polymers is detailed out individually to highlight potential factors that may enhance recycling. Additional information asked to the plants focused on other ‘side-stream’ fractions such as foreign polymers, polyolefin agglomerates, or foreign materials such as labels/closures, but could be obtained only for one plant. This information is therefore not provided.

The next sections provide additional details on the methodology followed for the collection of data and calculations of recovery rates alongside presenting process flows and operational parameters of the surveyed plants. In addition to the overview of the surveyed plants presented in Table 1, detailed information about the type of input-waste, challenges and opportunities to improve, for each of the surveyed plants, are provided in the SI document. It should be noted, however, that specific data such as mechanical equipment and other operational parameters are commercially sensitive and thus not presented.

#### 2.2.1 Material recovery facilities (MRFs)

**Fig. 2:**
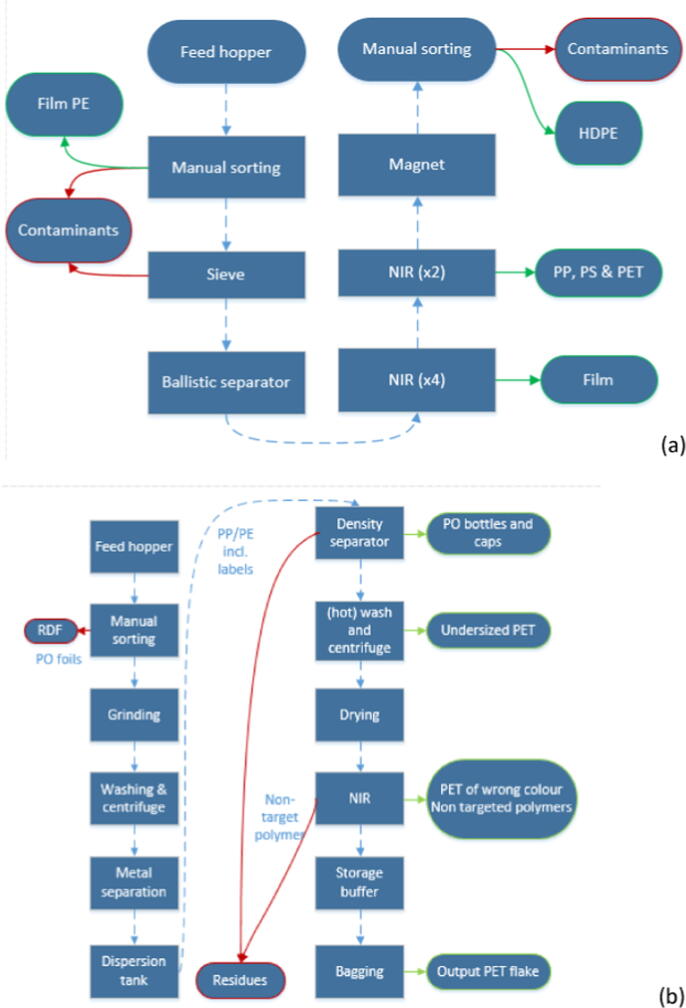
Process flow charts of the plants MRF1 (a) and REC5 (b); the dash line shows the process flow, residues and RDF streams are depicted with red, output streams of the plant are marked in green. Abbreviations may be found in the dedicated section. MRF: Material recovery facility; NIR: near infra-red scanner; REC: recycling facility.

The five surveyed MRFs receive light packaging waste from EPR systems and produce bales of sorted polymers (cfr. Table 1). Typically, the MRFs consist of five processes: feeding and pre-conditioning, conditioning, sorting, refining and product handling (Cimpan et al., 2016). The technological equipment varies across the plants and usually is subject to a trade-off among the processing costs, market demand, material prices and quality specifications of the output materials. The process flow of the MRF1 is illustrated in Fig. 2a, as an example. The sorting rate was calculated based on the average annual operations including technical failures and periods of working under-capacity (Eq. (1)). The background data used (input/output streams) are reported in Table S1 (SI). For each of the surveyed sorting plant, information on the purity rates of the output recyclable materials was also collected.

#### 2.2.2 Recycling plants (RECs)

The eight plastic surveyed RECs receive MRF-sorted PPW and target different output materials (cfr. Table 1). Fig. 2b depicts the flow chart of REC5, a PET recycling plant, as illustrative example. Similarly, mass flows and purity of the recyclates as well as information about the barriers that hinder enhanced recycling of PPW were collected through a detailed questionnaire. In addition to the information in Table 1, detailed mass balances, challenges and opportunities to improve can be found in the SI. The recovery rate was calculated based on the average annual operations including technical failures and periods of working under-capacity (Eq. (2)). The background data used (input/output streams) are reported in Table S1 (SI). Notice that some of the recycling plants perform additional sorting prior to actual recycling, whereas others receive already pre-sorted materials.

### 2.3 Material flow analysis of post-consumer PPW in EU27

The software STAN (Cencic and Rechberger, 2008) was used to elaborate two EU27-wide MFAs for PPW for years 2017 and 2030, striving to represent the *status quo* and *future targets* scenarios. The MFAs included the following waste management processes: generation, collection, sorting and recycling (cfr. Fig. 1), where the output of one process becomes the input of the following one. Within each process, the outputs equal to the sum of inputs multiplied by their transfer coefficient (TC).

#### 2.3.1 Modelling the status quo (scenario for year 2017)

The amount of PPW generated in EU27 in year 2017 was calculated departing from the latest available data (2016; 16,690 kt) assuming a 2.4% growth rate based on the average of the 2013–2017 generation rates (Eurostat 2020), equalling 17,080 kt a^−1^. The composition of PPW was based on PlasticsEurope 2017 (Table S2; 18% PET, 20% HDPE, 32% LDPE, 20% PP, 7% PS, 3% PVC; data for LLDPE and EPS are included in LDPE and PS values, respectively). The separately collected PPW was assumed to be mechanically recycled via MRF sorting and recycling plants, producing a secondary raw material for each target polymer. The non-captured PPW (i.e. collected with the mixed waste) was sent to final disposal (incineration or landfill), together with the rejects (losses) from the sorting and recycling stages. An average EU configuration of the recycling processes was assumed. The capture rates (used as TC in the collection phase) were based on the figures reported in Deloitte et al. (2017), and varied between 20% and 62% (Table S3). The sorting and recycling rates (used as TC in the sorting and recycling phases) represent the median of the datasets used. Such datasets (presented in Table 2 for each of the plastic polymers) were composed of primary data from this study (described in full in Section 3.1) complemented with data available from literature studies that collected primary data from EU facilities (WRAP, 2019, Brouwer et al., 2018, Conte, 2016, Eule, 2013, Mastellone et al., 2017; and Van Eygen et al., 2018) to ensure a maximum of representativeness across EU. The standard deviation was calculated assuming a triangular distribution of the dataset, because of the diversity of the information within the dataset. Finally, it was considered that in 2017 the amount of sorted material (*post* MRF) exported outside EU equalled ca. 152 kt month^−1^ (mainly to Asia; EEA, 2020), i.e. 1800 kt a^−1^.

**Table 2:** Sorting and recycling rates of target materials at the MRFs and RECs surveyed in the study. For the purpose of comparison, we report the figures from selected literature studies that provide primary data from full-scale operating plants in EU. Average, median, 75% percentile and standard deviation (TD: triangular distribution was assumed) are calculated on the basis of the primary and literature data listed in the Table. These parameters are the ones used for the material flow analysis modelling. MRF: material recovery facility; REC: recycling plant; NA: not available.

**Plant ID/Reference**	**PET**	**PP**	**PS**	**Films**	**HDPE**	**PVC**
	Sorting rate					
MRF1	45%	31%	31%	55%	95%	
MRF2	84%			72%	87%	
MRF3		80%	65%	60%	85%	
MRF4			25%	18%		
MRF5	97%			89%	13%	
Eule (2013)	94%			73%	86%	
Mastellone et al. (2017)	89%	7%			100%	
Van Eygen et al. (2018)	72%	44%	37%		53%	
WRAP (2019)	91%	91%	79%	85%	98%	73%
Conte (2016)	62%	78%		21%	64%	
Brouwer et al. (2019)	86%	68%		59%	77%	
Brouwer et al. (2018)	84%	60%		49%	80%	
Average	81%	57%	47%	58%	76%	73%
Median^a^	85%	64%	37%	59%	85%	73%
75% percentile^b^	91%	79%	65%	73%	91%	73%
Standard deviation (TD)	11%	18%	12%	15%	19%	NA

	Recycling rate					
REC1		53%		55%		
REC2	66%					
REC3	63%					
REC4	70%				70%	
REC5	87%					
REC6		60%			72%	
REC7	90%	72%		50%	88%	
REC8		85%	75%			
WRAP (2019)	75%	56%	57%	71%	87%	80%
Brouwer et al. (2019)	95%	95%		94%	95%	
Brouwer et al. (2018)	93%	86%		86%	94%	
Average	80%	71%	66%	71%	84%	80%
Median^a^	81%	66%	66%	71%	88%	80%
75% percentile^b^	91%	85%	71%	86%	93%	80%
Standard deviation (TD)	7%	9%	4%	9%	5%	NA

a main value used in the “status quo” MFA scenario.

b main value used in the “future targets” MFA scenario.

#### 2.3.2 Modelling future targets (scenario for year 2030)

The scenario for year 2030 models the way European post-consumer PPW is expected to be handled as a result of the efforts to meet the 55% recycling target by 2030. The configuration of the waste management system was assumed as in the *status quo* scenario, also using an annual 2.4% growth rate to forecast the generation of PPW in EU27 in year 2030. This assumption enables to single out the effects from the logistic, technological and behavioural improvements in the recycling of PPW that are needed to reach the 2030 targets. Such improvements were implemented in the model by using, for the capture rates, best practices as identified by Dri et al., 2018, Tallentire and Steubing, 2020 (69% was used). For the sorting and recycling rates, the 75% percentile of the dataset in Table 2 was used (as opposed to the median used in the *status quo* scenario), to reflect best available technologies. The whole dataset was still used to calculate the uncertainty around the values. The 2030 scenario considers that the entirety of the amount of sorted PPW (*post* MRF) is recycled domestically in the EU, given the high uncertainty on how exports to Asia will develop in the future and the EU intention to limit waste shipping.

## 3 Results and discussion

### 3.1 Material recovery and purity rates

The MRF sorting rates varied depending on the target polymer (Table 2). The highest sorting rate was observed for HDPE and PET; yet, a wide variation was observed across the plants (13–95% for HDPE and 45–97% for PET). The highest rate was observed in plants MRF1 for HDPE and MRF5 for PET, due to the presence of an efficient pre-sorting stage. For PP, the rate varied between 31% and 80%, with the highest efficiency observed for plant MRF3 due to the use of emerging sorting technologies (e.g. a high definition camera to identify contamination) and that the sorting line has multiple points at which missorted items are captured and redirected to the appropriate point in the sorting process. Also, differently than the remaining MRFs, a quality control on the residual fraction is carried out in MRF3, which results in increased sorting rates. For the same reason, the highest sorting rate for PS (65%) was achieved by plant MRF3. In contrast, the lowest yield for PS was observed in plant MRF4 (25%) owing to technological limitations e.g. a minor width of the conveyor belt that causes a high amount of rejects likely including target fractions. In respect to films, the sorting rate ranged from 55% to 89% with the highest rate observed for the plant MRF5. Plant operators reported that a manual sorting at the beginning of the sorting line allows them to collect large amounts of films with high purity rates (MRF 3–4; Table 3).

**Table 3:** Purity rates of the target output materials of the plants surveyed in the study. MRF: material recovery facility; REC: recycling plant; NA: not available.

**Plant ID**	**PET**	**PP**	**PS**	**Films**	**HDPE**
MRF1	NA				
MRF2	95.5%			82%	
MRF3	98%	98%	98%	98%	98%
MRF4	65%	92%	90%	92%	
MRF5	98%				98%
REC1				97%	
REC2	98%				
REC3	97%				
REC4	97%				
REC5	98%				
REC6	97%				
REC7	80%	80%			
REC8		95%	95%		85%

The sorting rates of the surveyed MRFs were found to be generally aligned with those reported in comparable literature (Table 2). Exceptions were the sorting rates for PS in MRF4, which is much lower than values reported by Faraca and Astrup, 2019, Cimpan et al., 2016. MRF4 operators reported that the low rates occur mostly due to the presence of multilayer and biopolymers in the input material, which affects the sorting rate. Similarly, sorting rates for PET in MRF1 appeared to be very low, mainly due to the very poor quality of the input materials coming from a commingled-streams separate collection, which is known to compromise recovery rates due to higher levels of contamination. Moreover, MRF1 targets individual streams of PET bottles and PET trays. This is achieved by separating first the PET fraction from the other polymers and streams and then carrying out a sorting between trays and bottles. This operation results in relatively low yield and overall high operating costs. MRF3 and MRF5 operators also reported that the presence of volatile compounds in the input materials can hamper significantly the sorting rates, experiencing a decrease of up to 30%.

In general, when comparing our results with the literature, it should be borne in mind that the figures reported in Eule, 2013, Mastellone et al., 2017 were obtained from dedicated sampling carried out at the facilities under controlled conditions, while the data we collected in this study are based on the average annual operations including technical failures and periods of working under-capacity. A similar approach was adopted by Van Eygen et al. (2018), whose average annual Austrian figures indicate overall lower performances than Eule (2013) or Mastellone et al. (2017), in line with our findings. Plant operators reported that all the produced bales typically comply with the output quality specifications set by the EPR system via the PRO. However, some plant operators reported that for certain output materials higher impurities are accepted by the market. For instance, for sorted PP a 10% impurities is accepted by the market, and up to 20% for films (see Table 3; plants MRF2 and MRF4). For PET, the plant MRF4 reported a purity of 65%, due to the presence of small films (i.e. fines) which cannot be removed efficiently throughout the process. For the plant MRF1, data on purity rates of the outputs were not available as the national PRO has not established quality specifications.

Three of the surveyed RECs focus their operations only on PET, while the remaining target three to four different polymers. The recycling rates varied greatly depending upon the type of polymer considered. The decision on the target polymers is essentially made upon the market demand and prices of the recyclates, as well as the purity of the streams of the input material. In operational terms, based on the above mentioned factors, the operators select which polymers and/or non-target materials to be selected and/or ejected by the NIR respectively. The recycling rate reported by the plants was 70–88% for HDPE, 63–90% for PET and 53–85% for PP (Table 2). The lower rates observed in some plants for PET (REC3) and PP (REC1) were linked by the plant operators to contamination of the input material (i.e. presence of volatile components such as acetaldehyde and benzene). For PS and films the reported recycling rates were 75% and 50% (REC8 and REC7), owing mostly to ineffective removal of additives and/or ink added to the original material, which often results in discoloured recyclates.

The reported purity of the output streams varies between 80% (PET and PP; REC7) and 98% (PET; several plants), as indicated by Table 3. All surveyed plants (MRFs and RECs) have in place output quality specifications, established by the various EPRs, except for MRF1 where the operators aim to achieve purity rates that are generally accepted by the market. Typically, the produced recyclates by all surveyed plants comply with the output quality specifications in respect to purity as set by the EPR system. However, it was reported by some plant operators (e.g. REC7) that, specifically for PP, the market can accept materials with slightly higher impurities (appx. 5% more than what requested by the output quality specifications).

### 3.2 Barriers and problems that limit recycling and quality in the investigated plants

Table 2 shows a relatively high variation of the sorting and recycling rates of the surveyed plants. According to most of the plant operators, this is typically related to technological limitations and quality of the input material. Regarding the second factor, the commingled collection of recyclables incurs higher contamination while also hampering normal operations. A common issue in all surveyed plants was the presence of films in the input material. Various plant operators (e.g. MRF2, MRF3, REC1) reported that when films are not removed effectively at the pre-sorting stage (like in plant MRF5), then these could entangle easily on moving parts and cause blockages, thus affecting the normal operation of the plant. Likewise, the flexible post-consumer packaging gets often stuck in the various sensors affecting the sorting processes. The improvement of the technical yield could take place only by improving lines with dedicated NIR detectors as stressed also in Mastellone et al. (2017). Hahladakis and Iacovidou (2019) highlighted that contaminants might not be completely removed at pre-sorting and washing stages, thus complicating the subsequent mechanical recycling stages. REC3 operators reported as well the presence of low amounts of hazardous waste and gas canisters in the bales received by the plant as well as other low quality (value) smaller films/materials, e.g. fines, multilayer. Especially for the multilayers, the main problem is to find appropriate markets since this type of material can be a valuable feedstock for chemical recycling applications (Ragaert et al., 2017). Additionally, MRF5 operators mentioned that UV protective additives as part of multilayer PET bottles are difficult to detect but cause unwanted stain, colour switch and melting point problems in the subsequent PET recycling. Therefore, the plant operators generally stockpile the collected amount of multilayers. Similarly, MRF3 and MRF4 operators communicated that non-packaging PP and PE items can successfully pass polymer detection stages due to their main material matrix but they may contain unwanted content, e.g. toner cartridges and composite materials or as blends with other non-polyolefin plastic (Ragaert et al., 2017). Another barrier for the sorters is represented by the presence of PET-based textiles in the input recyclables. In fact, the PET-based textiles are detected and successfully sorted by the NIR sorters but constitute impurities in the output-products. Offtakers typically decide whether the amount of these impurities can be accepted based on the type of final application.

We observed that the operational manner of the plant also influences the quality of the recyclates and the plants’ overall recovery rates. In fact, the actual throughput of MRF2, REC1 and REC7 plants exceeds the designed (nominal) capacity thus affecting the recovery and purity rates of the output materials. This issue was also stressed as recurrent in the review conducted by Cimpan et al (2016). In contrast, MRF1 operators noted that when most of the input materials arrived bagged to the plants, then the throughput decreased. This could be solved by upgrading the speed and power of the bag splitter. REC7 operators reported that at the end of the sorting line a dedicated NIR is installed to capture any PET, PP and HDPE fraction missed during the previous sorting steps. These materials are recirculated so the polymer specific optical sorters can capture it when economically convenient. Indeed, REC7 operators explained further that the recirculation of these collected materials is performed only under favourable market conditions. Plant operators (e.g. REC8 and MRF2) reported that the size of packaging is another barrier. Small size bottles or containers < 50 mm fall through the holes of the trommel ending up in the rejects. They further reported that the risk of presence of plastic foil and film still contained in the PP input to the plant could be mitigated by increasing the amount of polyolefin agglomerate fraction. This is technically feasible by installing a more efficient conveyor belt system at the air sifting section. With respect to the belt, MRF4 operators reported that installation of wider belts results in higher recycling rates for most recyclates.

With respect to HDPE, the main challenges for distributing it in the market and using it in higher value applications are the residual odours and the lack of colour separation (for the majority of the surveyed plants). The colour separation contributes to increase its value due to the strong market demand for natural and white HDPE from the packaging sector. However, this varies considerably across the EU, since certain colour fractions have specific applications, e.g. manufacture of new bleach bottles from yellow bottles. Likewise, separating transparent PET from a mixed colour PET fraction will most probably make the mixed PET fraction darker, which results in a lower sales value than lighter coloured mixed PET (with a high transparent PET content).

Regarding PP, the operators reported that it is challenging to obtain its colour separation with a high yield and purity, in line with the analysis of Mastellone et al. (2017). In this respect, certain operators send the recovered PP fraction to other plants for colour separation in order to use the PP recyclate in lighter coloured applications. MRF2 operators reported that the non-sorted PP fraction ends up in the mixed plastic fraction and/or recovered along with any residual HDPE/LDPE fractions, i.e. sold as mixed plastic fraction (typically as fuel). With respect to PS, it was reported by most of the plant operators that the market for this fraction is not mature yet. The high impact polystyrene sheet can be used in food packaging, however recycled pellets of PS are not of sufficient quality to be used in sheet applications and do not meet food contact standards. REC8 operators pointed out that their recycled PS is suitable for the manufacturing of coat hangers. Finally, a common problem was observed by almost all recycling plants in the density separation stage. In fact, the presence of certain fillers in items such as lids and caps make them sink instead of floating.

All the surveyed plants have in place protocols for quality management. Input materials are usually tested by the supplier whereas for the output materials laboratory tests are carried out. Therefore, the operators are able to know the composition of all the produced bales/recyclates but also to obtain information about the effectiveness of their sorting and recycling processes in real-time. Therefore, they are able to know as well the level of losses of certain polymers to other streams throughout the various sorting and recycling processes. Despite the fact that very few operators out of the surveyed plants target colour-separated polymers, the quality-related information about colour-separated polymers was further assessed in Table 4 to highlight the improvement potential. In this respect, Table 4 lists the losses for PET and HDPE/PP to other fractions based on the primary information gathered from the surveyed plants.

**Table 4:** Losses for PET and HDPE/PP to other output fractions; synthesis of the primary information from the surveyed plants.

**Polymers**	**Losses to:**	**Level of losses**	**Remarks**
PET green bottles	other bottle fractions	Not observed	Relevant for plants with PET colour sorting
PET transparent bottles	other bottle fractions	Very low	Practically no improvement potential; maximum potential of mechanical and manual sorting has been reached; No market demand; would demand technical adjustment of the optical sensors at negative cost/benefit
PET light blue bottles			
PET bottles	RDF	Low	Very limited improvement potential; maximum potential of manual sorting has been reached; would demand technical adjustment of the optical sensors at negative cost/benefit
PET	RDF	Considerable	All PET trays end up in the RDF stream; not a big market for recycled PET trays (though REC3 plant has started to process PET trays); large improvement potential
HDPE/PP	PET	Not observed	–
HDPE/PP	RDF	Considerable	Improvement potential exists, economic benefits need to be thoroughly analysed

NB: level of losses is based on managers’ judgements of the rejects produced throughout the sorting and recycling processes

### 3.3 Material flow analysis of post-consumer PPW: *Status quo* and *future targets*

**Fig. 3:**
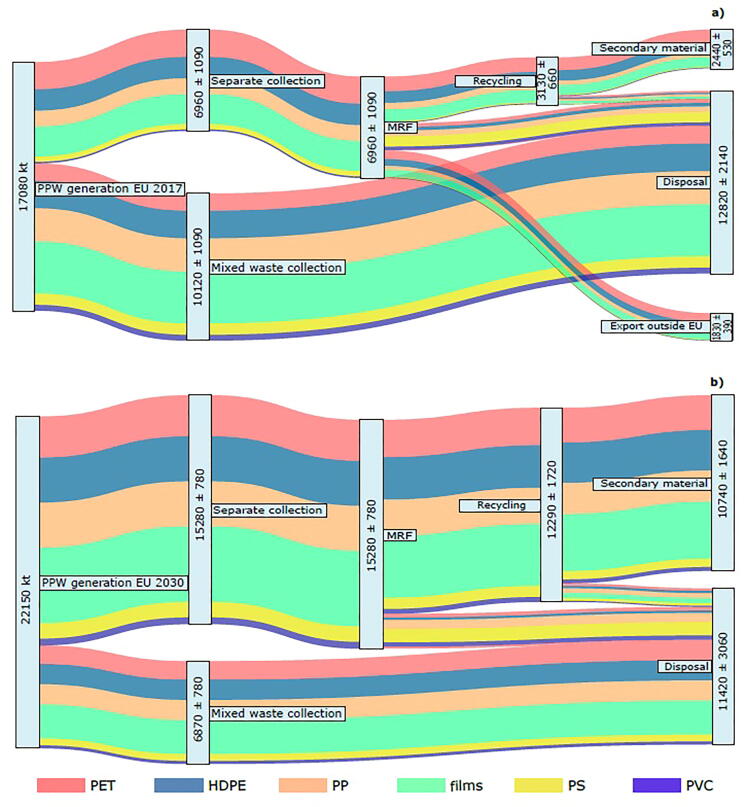
Material flow analysis of the EU27 post-consumer PPW (kt a^−1^); a) *status quo* (year 2017) scenario and b) *future target* (year 2030) scenario. The values reported refer to median ± standard deviation (confidence interval 68%). Different colours represent (from top to bottom): PET (red), HDPE (blue), PP (orange), films (green), PS (yellow) and PVC (purple). Values are rounded. MRF: material recovery facility; PPW: plastic packaging waste.

The results of the MFA illustrating the EU27 PPW management system in 2017 and 2030 are displayed in Fig. 3. According to our model, in 2017 around 29 ± 6% of the post-consumer PPW generated in EU27 was sent to recycling after sorting operations at the MRFs. This figure takes into account both the non-captured PPW that does not enter the recycling value chain and the rejects occurring at the MRF. The non-captured PPW represented around 60% of the PPW generated that, due to the type of collection in place, the specific guidelines set by PRO and the consumer behaviour, ended up disposed of directly via incineration or landfilling. On the other hand, sorting losses at the MRFs amounted to around 29 ± 12%, reflecting the barriers and bottlenecks discussed in Section 3.2. Not all materials in output from the MRFs are currently treated within the EU27 borders: we estimated that 37 ± 6% of the sorted material in 2017 was shipped off EU27 for utilisation in non-EU countries. Given the difficulty in tracking non-EU waste management systems and the long and fragmented recycling chain (involving many actors and operators), it is expected that only a share of the plastic exported is ultimately recycled. If we assumed that the exported PPW does not contribute to the EU27-wide recycling rate, the amount of recycled plastic in 2017 equalled 14 ± 3% of the PPW generated. If the exported waste is included in the calculation, the overall end-of-life recycling rate equals 25 ± 6% and is comparable to the ca. 30% estimated by Van Eygen et al. (2017) for Austria and Brouwer et al. (2018) for the Netherlands. It is important to bear in mind that all mass flows in Fig. 3 include impurities (to a certain extent, as acceptable by the market and/or subsequent receiving plants), according to Section 2.1. This is also true for the “secondary material” flows (see Eq.2), whose typical purity rates can be found in Table 3.

When logistic, technological and behavioural efforts are undertaken to increase capture, sorting and recycling rates, as it is expected in the mid-term future (*future targets* scenario), the PPW recycled into secondary raw material increases significantly (Fig. 2b). The amount of material sent to recycling equalled 55 ± 8% of the PPW generated, almost doubling the values of 2017 due to the large decrease in sorting losses at the MRF (20 ± 10% in 2030) in line with the expected technological improvements. Finally, the amount of PPW expected to be recycled in 2030 equalled 10,740 ± 1640 kt corresponding to an overall end-of-life recycling rate of 49 ± 8% (70 ± 11% of the PPW collected is ultimately recycled), under the assumption that all generated waste is treated domestically within the EU borders.

### 3.4 EU recycling targets: Where do we stand?

When comparing the MFA results for 2017 and 2030 (Fig. 3) with the target set in Directive (EU) 2018/852 (55% by 2030 for PPW; European Parliament and the Council, 2018), it is clear that large efforts are needed to achieve such an ambitious target, in line with what suggested by Van Eygen et al., 2018, Eriksen et al., 2020. According to the Directive, the formula to calculate the recycling target considers the amount of material that enters the actual recycling stage, i.e. losses due to additional sorting steps, e.g. float-sink separation even if done at the recycling plant, must be accounted for. The results of our model suggest that the 2030 PPW recycling rate could be between 49% and 55% (41–63% including the uncertainty associated with the results), depending on how sorting of impurities is accounted for, provided the adoption of measures simultaneously targeting the collection and the sorting/recycling technologies. It is worth noticing that the technology efficiencies used in the modelling represent the 75% percentile of those already performed by some plastic recycling plants in Europe (cfr. Section 3.1), suggesting that it may be realistic to enhance the current levels of sorting and recycling technologies. However, it is to be borne in mind that economic viability and market factors were not considered in our modelling, although these are expected to play a constraining role (Overgaard et al., 2018, Rigamonti et al., 2018). Moreover, the model did not capture the possibility that plastic recyclates may only be suitable for some (lower quality) material applications, e.g. coat hangers, textiles, pipes, outdoor benches; secondary plastic uptake may be limited once the market for these product categories become saturated, unless the expected technological improvements will cover also quality-related factors (e.g. colour, odour, presence of chemicals) in addition to yield increases. However, it is expected that mechanical recycling of PPW would be complemented by chemical recycling technologies, which would enable restoring the plastic monomer, while simultaneously separating it from impurities, thus further contributing to increasing PPW recycling rates.

### 3.5 Factors enabling higher quality recycling for PET, PP and PE

As mentioned in Section 3.4, further improvements are essential in the entire recycling chain to meet the PPW recycling target for 2030 i.e. collection, sorting and recycling. This is challenging because collection, recycling and sorting are often carried out by different actors with different interests resulting in additional complexity in the plastic material loops (Dri et al., 2018, Hahladakis and Iacovidou, 2019). The analysis of the barriers (Section 3.2) showed that, to be effective, improvements should address decisions made upstream of the entire recycling chain, such as product design, i.e. the recycling-oriented choice of packaging materials at the design phase, and collection systems. At the same time, factors such as available and emerging markets of each different polymer, process operational characteristics, available recycling technologies and policies applied can very much influence the efficiency of the entire recycling chain.

A list of potential improvements was developed, based on the quality-related information from the surveyed plants, for PET, PP and PE only, as these are the main polymers for which recycling technologies and secondary market are feasible (Table 5). Plant operators shared with PET materials can be recycled several times; however, PET recyclates purity can be enhanced when all beverage PET bottles are extracted as early as possible in the recycling chain to avoid contamination and further processing costs (Eriksen et al., 2019). Moreover, PET bottles when colour separated achieve rather high efficiency rates and are suitable to be used safely in the manufacture of food contact packaging. This, combined with the high market price of the PET recyclate when compared to the virgin PET, and the strong social pressure for purchase of beverage bottles with full or large content of recycled PET, enables optimal economic conditions for high quality recycling. However, the presence of multilayers, especially in PET trays, makes sorting rather challenging.

**Table 5:** Potential improvements for PET, PP and PE (LDPE and HDPE) that could enable high quality recycling; based on the quality-related information from the surveyed plants.

**Factors**	**PET**	**PP and PE (LDPE and HDPE)**
Operational	Sorted into a sufficient quality for the production of recycled PET for bottle to bottle manufacture Colour separation and recyclates used in food contact packaging	Improvements are essential in plant process design (see section 3.2)
	High impermeability that allows the production of recycled PET with low levels of contaminants and presence of odours, compared with other polymers	
Market	High demand for high quality materials from beverage bottle manufacturers	Development of additional markets for lower value fractions
Collection	Deposit Refund Scheme (DRS) in place for PET bottles; this limits the amount of non-PET bottles and also reduces the processing costs and ensures the homogeneity (Eriksen et al., 2019)	–
Recycling technologies	Not observed, recovering rates do not depend on the technologies employed	Improvements are possible but are subject very much to the market factor above
Recyclates' prices	Prices for food-grade recycled PET polymer are high whereas the price of virgin polymer is lower, since manufacturers purchase recyclates	–
Product design	–	Improvements in the choice of packaging materials and design-for-recyclability are essential (also stressed in Eriksen and Astrup, 2019)

In contrast to PET, recycling of PE and PP is rather challenging. In respect to PE, the challenges that can ensure high quality of recycling apply both to HDPE and LDPE as highlighted by Gala et al., 2020, Hopewell et al., 2009, and Horodytska et al. (2018). PE and PP fractions have a high degree of heterogeneity and their recycling usually lead to lower quality products, as also observed by Eriksen and Astrup (2019). On top of a larger variation in terms of products as compared to PET (e.g. compounded polymers, colour pigments, sizes), PE and PP degrade consistently during the use phase. This constitutes a limiting factor because materials tend to break up into particles of smaller size and end up lost into the fines fractions. On top of this, the secondary markets for these polymers are limited compared to PET. However, the total converters demand for PP and PE is way larger (around 10 and 6 Mt in EU28 + NO/CH in 2018, respectively) than PET (4 Mt; PlasticsEurope, 2020).

## 4 Conclusions

This study collected primary data from plants sorting and recycling plastic packaging waste to illustrate process efficiencies, material flows, and barriers. While all the produced outputs by all surveyed plants met the output quality specifications, or those typically accepted by the market, significant losses of target materials nevertheless occurred both at sorting and recycling stages. These were higher for polymers such as polypropylene and polystyrene, and lower for polyethylene terephthalate and high-density polyethylene. The overall end-of-life recycling rate for post-consumer plastic packaging waste in EU27 in 2017 equalled ca. 14% when the waste exported outside EU was not considered (25% when assuming that all the waste exported was also reprocessed). An improved scenario for 2030 showed that reaching an end-of-life recycling rate of 49% was possible, when best available practices and technologies were implemented. To fulfil the ambitious recycling targets set at EU level (55%), notable improvements are necessary at the plants, product design, collection system and market level. Our findings principally indicate that films and other problematic contaminants in the input materials considerably hamper the recovery rates, thus the improvement of the efficiency of the collection systems is imperative. In parallel, the development of markets for lower value fractions, e.g. polypropylene, could be a way forward to increase recycling, while improvements in the product design will reduce considerably the presence of impurities and contaminants in the input materials.

## 5 Disclaimer

The views expressed are purely those of the authors and may not in any circumstances be regarded as stating an official position of the European Commission.
