# Supplementary material for: Recycling of post-consumer plastic packaging waste in the EU: Recovery rates, material flows, and barriers
Source: Waste Manag. 2021 May 1;126:694–705. doi: 10.1016/j.wasman.2021.04.002 (PMC8162419; doi:10.1016/j.wasman.2021.04.002)
Supplement: Supplementary Data 1 [file mmc1.docx]

**Supplementary Information**

**Recycling of post-consumer plastic packaging waste in the EU: Recovery rates, material flows, and barriers**

Ioannis Antonopoulos*, Giorgia Faraca, Davide Tonini

European Commission Joint Research Centre, Calle Inca Garcilaso 3, 41092 Sevilla, Spain

*Corresponding author

This Supplementary Information (SI) document contains additional information in respect to: i) plants investigated, ii) information collected with the survey, and iii) assumptions taken and calculations performed to derive the material flow analysis (MFA).

Contents

[1. Methodological approach 4](#_Toc69377035)

[2. Plants overview 5](#_Toc69377036)

[2.1. MRF1 5](#_Toc69377037)

[Factors and parameters affecting quality of outputs 5](#_Toc69377038)

[Opportunities and barriers to improving quality of outputs 5](#_Toc69377039)

[2.2. MRF2 6](#_Toc69377040)

[Factors and parameters affecting quality of outputs 6](#_Toc69377041)

[Opportunities and barriers to improving quality of outputs 6](#_Toc69377042)

[2.3. MRF3 7](#_Toc69377043)

[Factors and parameters affecting quality of outputs 7](#_Toc69377044)

[Opportunities and barriers to improving quality of outputs 7](#_Toc69377045)

[2.4. MRF4 8](#_Toc69377046)

[Factors and parameters affecting quality of outputs 8](#_Toc69377047)

[Opportunities and barriers to improving quality of outputs 8](#_Toc69377048)

[2.5. MRF5 9](#_Toc69377049)

[Factors and parameters affecting quality of outputs 9](#_Toc69377050)

[Opportunities and barriers to improving quality of outputs 9](#_Toc69377051)

[2.6. REC1 10](#_Toc69377052)

[Factors and parameters affecting quality of outputs 10](#_Toc69377053)

[Opportunities and barriers to improving quality of outputs 10](#_Toc69377054)

[2.7. REC2 11](#_Toc69377055)

[Factors and parameters affecting quality of outputs 11](#_Toc69377056)

[Opportunities and barriers to improving quality of outputs 11](#_Toc69377057)

[2.8. REC3 12](#_Toc69377058)

[Factors and parameters affecting quality of outputs 12](#_Toc69377059)

[Opportunities and barriers to improving quality of outputs 12](#_Toc69377060)

[2.9. REC4 13](#_Toc69377061)

[Factors and parameters affecting quality of outputs 13](#_Toc69377062)

[Opportunities and barriers to improving quality of outputs 13](#_Toc69377063)

[2.10. REC5 14](#_Toc69377064)

[Impacts of markets and economic factors on plant operation 14](#_Toc69377065)

[Factors and parameters affecting quality of outputs 14](#_Toc69377066)

[Opportunities and barriers to improving quality of outputs 14](#_Toc69377067)

[2.11. REC6 15](#_Toc69377068)

[Impacts of markets and economic factors on plant operation 15](#_Toc69377069)

[Factors and parameters affecting quality of outputs 15](#_Toc69377070)

[Opportunities and barriers to improving quality of outputs 15](#_Toc69377071)

[2.12. REC7 16](#_Toc69377072)

[Impacts of markets and economic factors on plant operation 16](#_Toc69377073)

[Factors and parameters affecting quality of outputs 16](#_Toc69377074)

[Opportunities and barriers to improving quality of outputs 16](#_Toc69377075)

[2.13. REC8 17](#_Toc69377076)

[Impacts of markets and economic factors on plant operation 17](#_Toc69377077)

[Factors and parameters affecting quality of outputs 17](#_Toc69377078)

[Opportunities and barriers to improving quality of outputs 17](#_Toc69377079)

[3. Input/output data on plants’ material flows 18](#_Toc69377080)

[4. Material Flow Analysis (MFA): Background data 19](#_Toc69377081)

[*4.1 Plastic packaging waste composition (year 2017)* 19](#_Toc69377082)

[*4.2 Plastic packaging waste collection (year 2017)* 19](#_Toc69377083)

[*4.3 Modelling the Status quo scenario (year 2017)* 20](#_Toc69377084)

[*4.4 Modelling the Future target scenario (year 2030)* 20](#_Toc69377085)

[*4.5 Handling uncertainty (Status quo and Future targets scenarios)* 20](#_Toc69377086)

[*4.6 Plastic Packaging Waste flows (MFA results)* 20](#_Toc69377087)

[References 23](#_Toc69377088)

# Methodological approach

Within the frame of the study, interviews and site visits in 13 plastic packaging waste recycling plants (5 sorting and 8 recycling plants), located in the EU were carried out. Data and primary information gathered from the surveyed plants were collected through a detailed questionnaire focusing on 2 pillars: i. understanding current operations and ii. opportunities to improve quality. Figure S1 displays in detail the structure of the questionnaire and provides information about the type of questions asked to plant operators.

*Figure S1: Structure of the questionnaire*

The study did not focus necessarily on the top performing plants in the EU in terms of yield and purity of recyclates but also to plants that aim to improve efficiency of their processes and eventually to move towards the production of recyclates with very low level of impurities. In addition, the selection of the surveyed plants took into account differences of collection methods applied e.g. commingled or single-stream and targeted polymers. More information about the plants such as location, source of input material received, collection methods, targeted polymers and outputs are available in section 2 below and in Table 1 of the main article.

# Plants overview

# MRF1

| Plant ID | MRF1 |
| --- | --- |
| Annual capacity | 20,000 t |
| Inputs | Municipal light packaging collections targeting:  Boards and packaging papers, plastic packaging (including films), aluminium and ferrous packaging, aseptic cartons, glass. |
| Source of inputs | Municipal light packaging collections from the PRO that includes:   - on-street bulk bins, - apartment bin stores - some door to door collections.   Municipalities are contracted by the PRO to collect the material. |
| Outputs | Mixed papers, OCC, PET (bottles and trays), HDPE, (PP/PS mix), LDPE (4 different colour grades), steel and aluminium packaging |

### Factors and parameters affecting quality of outputs

**Strengths**

- Existence of a pre-sorting stage to remove some contaminants.

**Weaknesses**

- High levels of contamination of the input recyclables: a small amount of contaminants of input is at the pre-sorting stage. The rest contaminants enter the sorting processes which results in lower overall plant efficiency.
- Municipalities contracted by the national PRO are not obliged to meet certain composition specifications on contamination.

### Opportunities and barriers to improving quality of outputs

- Volatile economic markets make it difficult a capital investment in order to improve quality of the outputs.
- Short-term contracts with the PRO.
- Reliant on the material revenues, which leads to market fluctuations.

# MRF2

| Plant ID / name | MRF2 |
| --- | --- |
| Annual capacity | 30,000–36,000 tonnes |
| Inputs | Municipal light packaging collections targeting:  plastic bottles, plastic packaging (pots, tubs and trays), ferrous and non-ferrous containers, aseptic cartons |
| Source of inputs | Municipal bring bank collections of mixed light packaging |
| Outputs | Plastics: PET bottles (mixed colours), PET trays, HDPE bottles (mixed colours), mixed plastics (PP, PS, PET, HDPE, LDPE, PVC), film  Metals: ferrous containers, non-ferrous containers & foil  Other: Aseptic cartons |

### Factors and parameters affecting quality of outputs

**Strengths**

- The produced outputs of the plants should meet the output quality specifications set by the national PRO.

**Weaknesses**

- No incentive from the output quality specifications to exceed the minimum requirements.
- The existing cooperation.
- The plant operates at full capacity and some times over it. The high rate of processing material affects the output quality.
- Collaboration of the plant operator, municipality and PRO.

### Opportunities and barriers to improving quality of outputs

- Separation of PP from the mixed plastic fraction.
- Run the equipment slower
- Colour sorting HDPE, particularly as there is a good market for natural and white HDPE from the packaging sector. Also, other colour fractions have specific applications (manufacture of new bleach bottles).

# MRF3

| Plant ID / name | MRF3 |
| --- | --- |
| Annual capacity | 28,000 tonnes |
| Inputs | Household dry recycling collections targeting: Plastic bottles, PTT, paper and board, metal containers, aseptic containers |
| Source of inputs | Mostly from municipal comingled kerbside collections, targeting light packaging and paper; some inputs from separate collections of plastics or paper |
| Outputs | Paper: Hard mixed paper, newspapers & magazines, corrugated cardboard, aseptic containers  Plastics: Clear PET, coloured PET, HDPE, PP, PS, PE film  Metals: Aluminium and ferrous cans |

### Factors and parameters affecting quality of outputs

**Strengths**

- Quality control of the residual fraction, only 3% is accounted for not captured material
- National PROs can adjust payments when output does not meet output quality. specifications.
- Efficient and modern equipment used.

**Weaknesses**

- Materials not coloured separated.
- Odours presence in HDPE outputs.
- Not totally emptied plastic bottles which make them heavy to be ejected by the NIR.
- High contamination in the mixed material input streams.

### Opportunities and barriers to improving quality of outputs

- Upgrade and renew of the sorting equipment.

# MRF4

| Plant ID / name | MRF4 |
| --- | --- |
| Annual capacity | 40,000 tonnes |
| Inputs | Municipal light packaging collections targeting: Plastic bottles, PTT, metal containers, aseptic containers |
| Source of inputs | Household mixed light packaging collections by national PROs, collected comingled in yellow bins; and increasing in sacks, and separate containers that target only plastic bottles |
| Outputs | PET (various grades), PS/PP, LDPE (various grades), LLDPE, HDPE (various grades), mixed plastics, metals, aseptic containers |

### Factors and parameters affecting quality of outputs

**Strengths**

- Communication campaign carried out by municipalities and PROs increased the quality of the input materials.
- The national PRO has put in place Regular, comprehensive checks and sanctions (when it is needed) to encourage quality specifications.
- Visual inspection of the input materials upon arrival. Loads with unacceptably high levels of contamination are subject to a surcharge.

**Weaknesses**

- Undetected metal items in the beginning of the sorting process may end up together with sorted plastics in the press.
- Presence of multilayer/polymer compoplants/blends, filler substances, biopolymers and other contaminants in input material.
- PVC contamination in the film stream.
- Strapping tapes entangle easily on moving parts and cause blockages.
- The presence of lithium batteries may cause fires in the processes.

### Opportunities and barriers to improving quality of outputs

- Colour and type separation of films

# MRF5

| Plant ID / name | MRF5 |
| --- | --- |
| Annual capacity | 15,000 tonnes |
| Inputs | Household light packaging collections targeting: plastic bottles, plastic packaging (pots, tubs and trays), ferrous and non-ferrous containers, aseptic cartons |
| Source of inputs | Household mixed light packaging collections; approximately 90% from street containers and bring banks, and the remainder from door-to-door sack collections in rural areas |
| Outputs | Plastics: PET bottles (three colour grades), HDPE/PP containers, film  Metals: ferrous, non-ferrous containers  Paper: mixed paper & board  Other: aseptic cartons, WEEE, RDF |

### Factors and parameters affecting quality of outputs

**Strengths**

- Manual sorting included in the process.

**Weaknesses**

- Changes in the input material composition e.g. multilayer products, colour mixtures, heavily compacted items affect considerably the plant's sorting efficiency.
- Large sized (<1.5 ltr) bottles that are made with an extra UV-protection layer from PC with other polymer layers cause colour changes in the regranulate in onward recycling.
- Presence of odours in the LDPE fraction makes it difficult to be placed in the market.

### Opportunities and barriers to improving quality of outputs

The operator provided the following views regarding opportunities to improve the quality of outputs:

- The installation of NIR and/or robotics has been considered, but the benefits of this choice were not clear. The implied costs along with the insufficient experience are the main barriers.
- Improvement of the quality of the input materials e.g. reduction of the arising of multi-layer PET bottles.
- Additional sorting (i.e. manual) of PP and PS fractions from the RDF output.

# REC1

| Plant ID / name | REC1 |
| --- | --- |
| Annual capacity | 24,000 tonnes |
| Inputs | Household plastic films recovered from sorting centres |
| Source of inputs | Household film input from multiple sorting plants, including EPR and municipal clients |
| Outputs | PE pellet |

### Factors and parameters affecting quality of outputs

The key factors determining the quality of the outputs that the plant currently produces are:

**Strengths**

- Visual inspection of loads upon arrival in the plant prior to sorting
- Bales are labelled and their content is traced throughout the recycling process.
- Hourly checking of the bulk density of the pellet produced from each extruder to ensure specifications are being met.
- Investment in pre-washing sortation to produce pellet suitable for use in blown film applications.

**Weaknesses**

- Pellets are often discoloured due to the impurity presence. These impurities can be inks for instance that they were not removed throughout the recycling process.
- Further extrusion may be needed in pellets when metallised films have not been removed in the previous process steps.
- Long period of storing of films at sorting centres may increase the risk of getting food contamination and thus cause odour issues.

### Opportunities and barriers to improving quality of outputs

The operator provided the following views regarding opportunities to improve the quality of outputs:

- Production of a clearer pellet, without imperfections or black spots, would allow the plant to diversify with respect to the pellet’s end-use. This could be achieved through the introduction of colour sorting and the subsequent removal of heavily coloured film.
- A smaller screen could be integrated with the extruder to remove more non-PE plastic, again aiding in the removal of black spots from pellets.
- Using a hot wash instead of the current cold wash would further reduce imperfections.

# REC2

| Plant ID / name | REC2 |
| --- | --- |
| Annual capacity | 45,000 tonnes |
| Inputs | Baled mixed PET |
| Source of inputs | Baled mixed PET imported from Germany, collected via the DSD ('yellow bag') system |
| Outputs | Sorted Clear PET bottles, Coloured PET Bottles, Opaque PET, PET trays, mixed metals incl. aluminium and baling wire |

### Factors and parameters affecting quality of outputs

The factors driving higher quality outputs at the plant are:

**Strengths:**

- The speed of the sorting line
- Skills of the staff in the sorting line
- The range of suitable offtakers of quality of outputs available, and the higher prices paid by offtakers for higher quality outputs (compared with lower quality outputs).
- The requirements of the Waste Shipment Regulations, which influence the choice of offtakers and inform changes to on-plant processes.
- The requirements of downstream processors, who will be impacted if the quality of the outputs changes.

**Weaknesses:**

Not reported

### Opportunities and barriers to improving quality of outputs

- Increase of the recovery rates would be possible by moving to manual sorting to automated e.g. by installing NIR sorting (pending investment so far).
- Improve the design of the PET materials by eliminating any chemical protection or glue around the edges of the product. This would have positive effects in the quality of the output products.

# REC3

| Plant ID / name | REC3 |
| --- | --- |
| Annual capacity | Approx 62,000 tonnes (When both lines on bottles), Approx 50,000 tonnes (when tray line is processing trays) |
| Inputs | PET bottles and trays |
| Source of inputs | Multiple sources, primarily sorting plants and PROs located in the Netherlands, France, Germany, Belgium or the UK. |
| Outputs | rPET |

### Factors and parameters affecting quality of outputs

The key factors determining the quality of the outputs that the plant currently produces are:

**Strengths**

- Visual inspection of loads as they are offloaded from vehicles
- Incoming bales are sampled using a gravimetric test.
- Outputs are bale sampled using laboratory tests.

**Weaknesses**

- The rPET derived from trays has a lower IV than from bottles and therefore is not suited to bottle production. However, this material is suitable for trays.

### Opportunities and barriers to improving quality of outputs

The operator provided the following views regarding opportunities and barriers to improve the quality of outputs:

- The current value of rPET is sufficient that yield is the important factor. Therefore, the economics favour processing bottles rather than trays. If demand for rPET increases further, this may result in an increase in bottle prices therefore the economics of trays may improve.

# REC4

| Plant ID / name | REC4 |
| --- | --- |
| Annual capacity | 25,000 tonnes |
| Inputs | PET |
| Source of inputs | Mostly PET from EPR schemes and DRS collections, or from other sorting plants, in Austria, Italy, Slovenia, Croatia and Hungary. |
| Outputs | PET food grade, PET non-food grade |

### Factors and parameters affecting quality of outputs

The key factors determining the quality of the outputs that the plant currently produces are summarised below.

**Strengths**

- Due to industry recycled content commitments, there is a strong demand from brand owners for food contact recycled polymer.
- The market is good for food contact recycled PET, with high prices paid that for virgin PET or recycled flake.
- Downstream requirements necessitate an output purity of 95% - It is therefore paramount that the plant operator implements effective quality management and sorting technology.
- Reasonably extensive quality testing of outputs is carried out. Each bulk bag of PET flake has a sample taken from it and half of these are tested.

**Weaknesses**

- PVC & barrier layer PET result in the discoloration of the PET output.

### Opportunities and barriers to improving quality of outputs

- With respect to the main output of food grade PET, the plant operator does not consider that there is further room for improving quality.
- The company plans to install new equipment for processing the material that is currently ejected during the recycling to recover 9 fractions from this waste stream.
- There is a PO float fraction from the sink / float tank which the operator wishes to update to PO pellet for use in food contact applications, as there is strong market demand for this type of output.
- The plant operator considers that incentives should be in place within EPR schemes to encourage a more ‘closed loop’ system i.e. PET food grade bottles to PET food grade bottles, sheet to sheet etc. The plant operator is also in favour of fee modulation to encourage companies to place recyclable packaging on the market.

# REC5

| Plant ID / name | REC5 |
| --- | --- |
| Annual design capacity | 40,000 tonnes |
| Inputs | Pre-sorted PET, mostly containers, ~5% monolayer trays |
| Source of inputs | PET bottles mostly from DRS system; and household mixed light packaging plastic fractions DSD collections. |
| Outputs | PET flake, 7 different specifications |

### Impacts of markets and economic factors on plant operation

- Input material from other sources is purchased through a combination of long-term contracts and spot prices.
- The materials are sold by the plant operator on the open market.

### Factors and parameters affecting quality of outputs

- Installation of additional flake sorting equipment.

### Opportunities and barriers to improving quality of outputs

- All outputs are sold on the open market: i. possible financial benefit from obtaining higher prices for higher quality outputs, ii. risk of market prices falling for higher quality outputs. The operator is more concerned about long-term demand (rather than short term price fluctuations).
- Increased public demand for more environmentally sustainable management of plastics is a driver for improving the quality of outputs.
- Further decontamination and extrusion of PET flake would be uneconomical e.g. high energy use.
- Further improvement of purity of material is needed in collection.
- Avoiding complex designs in manufacture of PET products.

# REC6

| Plant ID / name | REC6 |
| --- | --- |
| Annual design capacity | 40,000–45,000 tonnes |
| Inputs | PP and PE, sorted bales |
| Source of inputs | Pre-sorted bales of PP and PE from MRF |
| Outputs | PP or HDPE granule (tailored to customer specification); High-purity reprocessed PP and HDPE blend granule (tailored to customer specification); Sorted Polyolefin Film Agglomerate (90–98% purity) |

### Impacts of markets and economic factors on plant operation

- A PRO charges the plant operator a price for the input material which is adjusted based on fluctuations in raw material markets; the output value/revenue is set at a fixed rate in the contract with the PRO. The plant operator does its own marketing of outputs on open markets through a separate business as well.

### Factors and parameters affecting quality of outputs

- The requirements of downstream processors purchasing outputs materials.
- Pursuit of higher cost-efficiency to increase revenues.
- The incentive to maintain leading market position.
- An ambition to stay compliant or ahead of ever more stringent safety and environmental protection standards.
- Investments in waste water treatment and water management systems improving the washing process and reducing drastically the odours of the produced granules.

### Opportunities and barriers to improving quality of outputs

- Marginal improvements are foreseen, such as replacement of intensive cold water washing with warm water (result in more efficient removal of impurities but also cause higher target polymer losses).
- The contracting of experienced and capable staff is a barrier.

# REC7

| Plant ID / name | REC7 |
| --- | --- |
| Hourly design capacity | 8 tonnes per hour (ca. 24,000 t/year) |
| Inputs | Mixed plastics |
| Source of inputs | Mostly from household collections of mixed plastics packaging from bring banks. PET bottles from DRS systems. |
| Outputs | PET, HDPE, PP, clear film, coloured film |

### Impacts of markets and economic factors on plant operation

- The plant receives a fixed contracted price per tonne of input material processed. Some contracted prices remain fixed for two to three years while others are subject to regular reviews, with prices typically fluctuating between a lower and upper bound. Adjustments to contracted prices are attributed to input tonnages over a baseline, rather than adjustments being made based on quality.
- The plant receives a fixed price per tonne of material produced to a quality standard. Sales are typically a commercial arrangement, and as such any notable change to market conditions usually coincides with an adjustment in prices, giving some flexibility. The most common arrangement is that a compounder buys flake or bales for a fixed price on a quarterly basis.

### Factors and parameters affecting quality of outputs

**Strengths**

- Eject fractions pass a control cabin prior to storage, allowing quality to be monitored.
- An NIR optical sorter is installed at the end of the sorting line, capturing any target material missed in the initial sorting process.

**Weaknesses**

- The plant operator is contracted to accept all materials placed in recycling containers by residents, whom themselves have no restrictions on the plastic packaging they can deposit in the containers.
- The presence of films particularly mixed large and small film, inhibits the performance of the plant’s optical sorters.
- In that country, a DRS for PET beverage bottles is in place, and therefore lower grade material such as non-food bottles and trays make up a larger composition of the plant’s PET inputs.

### Opportunities and barriers to improving quality of outputs

- More effective sorting at the material source, particularly by reducing the film contamination. (sorting technology for films is available).
- Methods to increase the recovery of the residual fraction could be explored since appx. 40% of the residual stream consists of target material i.e. HDPE, PP, PET.
- Higher quality flake could be obtained through use of a hot wash; however, for this change to be implemented the demand needs to exist in the market. The plant operator considers that the current market is not ready to pay the associated premiums.
- A further opportunity exists in producing mixed polyolefin flake from a mixed plastic input (but it is an expensive solution in the reporting period (2019)).

# REC8

| Plant ID / name | REC8 |
| --- | --- |
| Annual design capacity | 20,000 tonnes |
| Inputs | HDPE / PP / PS mix |
| Source of inputs | Multiple sources from various MSs: EPR collections. |
| Outputs | HDPE jazz/carbon, HDPE natural, HDPE opaque white, PP, PS |

### Impacts of markets and economic factors on plant operation

- Some input material is obtained via a fixed contracted price for each tonne of material processed.
- Other material is purchased at market rates from both municipal and EPR clients.
- The selling mechanisms are customer specific, with some of the recycled pellet sold at a fixed contracted price per tonne of material and to a quality standard, with other recycled pellet sold on the open market.

### Factors and parameters affecting quality of outputs

**Strengths**

- EPR systems ensure purity of the input bales appx. 95% purity.
- Visual inspection during offloading.
- Re-sorting of the residual fraction on a separate shift to recover PS, PP and HDPE which was missed in the first pass (although it is not targeted, PET is also recovered).
- Recovery and recycling of the fines fraction from the Trommel into a mixed PO pellet.
- Optimisation of the quality of the PS pellet by setting the optical sorter to recover only HIPS, not XPS or EPS.

**Weaknesses**

- Non-target contaminants that float along with target material when washed.

### Opportunities and barriers to improving quality of outputs

- The plant operator considers that the removal of odour from HDPE pellets, particularly natural HDPE pellets, could lead to an increase in demand from the packaging sector where odour is currently an issue. Similarly, the quality of HDPE and PP flake could be improved through the addition of a hot wash. In both instances, the technology to implement the process is readily available; however, the plant operator considers that the users of these materials are not yet ready to pay the associated premium required to make the investments viable. The plant operator envisions that this could change in the next one to three years.
- Some contamination exists in the PS pellets in the form of PO, as optical sorting is not 100% efficient and PO cannot be removed in a subsequent wash as it floats with PS. It is possible to sort the PP or HDPE flake from PS after washing and drying; however, due to their relative masses, there is likely to be an excessive yield loss of PS.
- The plant operator considers that there is a lack of consistency between EPR systems with respect to recovered plastics material specifications and that this is sometimes an issue. Each specification can require the sorting centres to grade in slightly different ways. European wide specifications therefore could prove beneficial.

# Input/output data on plants’ material flows

Table S1: Mass balance of the surveyed plants; absolute amounts of polymers or compositions were reported by the plant operators.

| **INPUT** | | | | | |
| --- | --- | --- | --- | --- | --- |
| Plants id | PET | PP | PS | Films | HDPE |
| **MRFs** | | | | | |
| MRF1 | 867 | 434 | 400 | 1301 | 108 |
| MRF2 | 248 |  |  | 309 | 3113853 |
| MRF3 | N/A – recovering efficiencies were given directly to the research team | | | | |
| MRF4 | 1740 | 1119 | 119 | 4330 | 700 |
| MRF5 | 30% |  |  | 5.70% | 29.70% |
| **Recycling plants** | | | | | |
| REC1 |  | 3.2 |  | 30.4 |  |
| REC2 | 111846 |  |  |  |  |
| REC3 | 63648 |  |  |  |  |
| REC4 | 25000 |  |  |  | 280 |
| REC5 | 35000 |  |  |  |  |
| REC6 |  | 30000 |  |  | 10000 |
| REC7 | 45% | 10% |  | 22% | 31% |
| REC8 | N/A – recovering efficiencies were given directly to the research team | | | | |
| **OUTPUT** | | | | | |
| Plants id | PET | PP | PS | Films | HDPE |
| **MRFs** | | | | | |
| MRF1 | 390 | 135 | 124 | 710 | 102.6 |
| MRF2 | 208 |  |  | 222 | 668946 |
| MRF3 | N/A – recovering efficiencies were given directly to the research team | | | | |
| MRF4 | 1290 | 300 | 30 | 770 | 260 |
| MRF5 | 29% |  |  | 5% | 4% |
| **Recycling plants** | | | | | |
| REC1 |  | 1.68 |  | 16.8 |  |
| REC2 | 7348.5 |  |  |  |  |
| REC3 | 39912 |  |  |  |  |
| REC4 | 17500 |  |  |  | 196 |
| REC5 | 30500 |  |  |  |  |
| REC6 |  | 18000 |  |  | 7000 |
| REC7 | 40.50% | 7.20% |  | 11% | 27.28% |
| REC8 | N/A – recovering efficiencies were given directly to the research team | | | | |

# Material Flow Analysis (MFA): Background data

## *4.1 Plastic packaging waste composition (year 2017)*

Table S2: Composition of the plastic packaging waste generated in EU27 in 2017, based on the figures reported in PlasticEurope (2017). The total for 2017 is estimated departing from the total amount generated in 2016 (16690 kt) assuming a 2.4% annual growth rate calculated from Eurostat (2020). PTTs: pots, tubes and trays (all rigid packaging excluding bottles and flasks).

|  |  | PET | HDPE | LDPE | PP | PS | EPS | PVC | LLDPE | **Total (kt)** |
| --- | --- | --- | --- | --- | --- | --- | --- | --- | --- | --- |
| Household Waste | Bottle | 61% | 36% | 0% | 2% |  |  |  |  | **3193** |
| Household Waste | PTTs | 31% | 12% | 0% | 56% |  |  |  |  | **2098** |
| Household Waste | Films | 2% | 12% | 69% | 16% |  |  |  |  | **3500** |
| Commercial & Industrial Waste | Bottle | 9% | 86% | 0% | 5% |  |  |  |  | **1289** |
| Commercial & Industrial Waste | PTTs | 27% | 32% | 0% | 41% |  |  |  |  | **1013** |
| Commercial & Industrial Waste | Films | 1% | 0% | 83% | 16% |  |  |  |  | **3188** |
| Others | Other pack. |  | 2% | 10% | 18% | 28% | 14% | 17% | 10% | **2799** |
| **GRAND TOTAL** |  | **18%** | **20%** | **32%** | **20%** | **5%** | **2%** | **3%** | **2%** | **17080** |

## *4.2 Plastic packaging waste collection (year 2017)*

Table S3: Capture rate for the individual polymers composing the plastic packaging waste in EU27 in 2017 based on the figures reported in Deloitte (2017); nr: not reported; PTTs: pots, tubes and trays (all rigid packaging excluding bottles and flasks).

|  |  | PET | HDPE | LDPE | PP | PS | EPS | PVC | LLDPE | **Total (kt)** |
| --- | --- | --- | --- | --- | --- | --- | --- | --- | --- | --- |
| Household Waste | Bottle | 79% | 76% | 0% | 32% |  |  |  |  | 76% |
| Household Waste | PTTs | 25% | 15% | 0% | 42% |  |  |  |  | 34% |
| Household Waste | Films | 0% | 12% | 37% | 18% |  |  |  |  | 30% |
| Commercial & Industrial Waste | Bottle | 36% | 40% | 0% | 18% |  |  |  |  | 38% |
| Commercial & Industrial Waste | PTTs | 51% | 13% | 0% | 65% |  |  |  |  | 44% |
| Commercial & Industrial Waste | Films | 0% | 0% | 39% | 32% |  |  |  |  | 38% |
| Others | Other pack. | 0% | 0% | 0% | 0% | 30% | 36% | 20% | 50% | nr |
| **GRAND TOTAL** |  | **62%** | **44%** | **36%** | **32%** | **30%** | **36%** | **20%** | **50%** | **40.8%** |

## *4.3 Modelling the Status quo scenario (year 2017)*

- - Polymer generation rates are based on secondary data (see Table S2).
  - Capture rates are based on secondary data (see Table S3).
  - Sorting and recycling rates are based on the primary data collected from the plants, further complemented with selected literature data; see Table 2 in the main manuscript (data used are “median” and “standard deviation (TD)”).

## *4.4 Modelling the Future target scenario (year 2030)*

- - Polymer generation rates are based on Table S2 considering an annual growth rate of 2.4% departing from the amount generated in year 2017.
  - Capture rates are based on best practices (Dri et al., 2018; Tallentire and Steubing 2020).
  - Sorting and recycling rates are assumed as the 75% percentile calculated on the dataset presented in Table 2 of the main manuscript (data used are “75% percentile” and “standard deviation (TD)”)

## *4.5 Handling uncertainty (Status quo and Future targets scenarios)*

The uncertainty related to the sorting and recycling rates was fed into the STAN software in order to take into account in the plastic waste flows the variability of such data. Given the small sample of data used, a triangular distribution was used. The uncertainty in the input data was then propagated throughout the model and took into account in the results, therefore obtaining results ranges, rather than exact values in themselves. This is why in Figure 3 results are reported as median ± standard deviation. Moreover, the model was reconciled, i.e. a statistical check was performed throughout the model to check whether results always stay within the 95% confidence interval.

## *4.6 Plastic Packaging Waste flows (MFA results)*

*Table S4: Plastic packaging waste flows (with uncertainty) for each modelled process in the 2017 and 2030 scenarios. Values are expressed in kt. The column “Value” represents the median value. The column “Uncertainty” represents the standard deviation.*

|  |  |  | **2017 - status quo scenario** | | **2030 - future targets scenario** | |
| --- | --- | --- | --- | --- | --- | --- |
| **Polymer** | **From** | **To** | **Value (kt)** | **Uncertainty (kt)** | **Value (kt)** | **Uncertainty (kt)** |
| PET | PPW generation | Separate collection | 1880 | 195 | 2736 | 140 |
| HDPE | PPW generation | Separate collection | 1460 | 213 | 2996 | 154 |
| PP | PPW generation | Separate collection | 1077 | 214 | 3011 | 154 |
| LDPE | PPW generation | Separate collection | 2075 | 360 | 5059 | 259 |
| PS | PPW generation | Separate collection | 373 | 74 | 1039 | 53 |
| PVC | PPW generation | Separate collection | 99 | 32 | 443 | 23 |
| PET | PPW generation | Mixed waste collection | 1177 | 195 | 1229 | 140 |
| HDPE | PPW generation | Mixed waste collection | 1888 | 213 | 1346 | 154 |
| PP | PPW generation | Mixed waste collection | 2288 | 214 | 1353 | 154 |
| LDPE | PPW generation | Mixed waste collection | 3579 | 360 | 2273 | 259 |
| PS | PPW generation | Mixed waste collection | 789 | 74 | 467 | 53 |
| PVC | PPW generation | Mixed waste collection | 396 | 32 | 199 | 23 |
| PET | Separate collection | MRF | 1880 | 195 | 2736 | 140 |
| HDPE | Separate collection | MRF | 1460 | 213 | 2996 | 154 |
| PP | Separate collection | MRF | 1077 | 214 | 3011 | 154 |
| LDPE | Separate collection | MRF | 2075 | 360 | 5059 | 259 |
| PS | Separate collection | MRF | 373 | 74 | 1039 | 53 |
| PVC | Separate collection | MRF | 99 | 32 | 443 | 23 |
| PET | MRF | Recycling | 1008 | 139 | 2490 | 248 |
| HDPE | MRF | Recycling | 783 | 168 | 2726 | 426 |
| PP | MRF | Recycling | 435 | 122 | 2379 | 403 |
| LDPE | MRF | Recycling | 772 | 186 | 3693 | 535 |
| PS | MRF | Recycling | 87 | 26 | 676 | 95 |
| PVC | MRF | Recycling | 46 | 15 | 324 | 17 |
| PET | MRF | Export to non EU | 590 | 82 |  |  |
| HDPE | MRF | Export to non EU | 458 | 99 |  |  |
| PP | MRF | Export to non EU | 254 | 72 |  |  |
| LDPE | MRF | Export to non EU | 452 | 109 |  |  |
| PS | MRF | Export to non EU | 51 | 15 |  |  |
| PVC | MRF | Export to non EU | 27 | 9 |  |  |
| PET | Recycling | Secondary material | 817 | 123 | 2266 | 257 |
| HDPE | Recycling | Secondary material | 689 | 151 | 2535 | 408 |
| PP | Recycling | Secondary material | 287 | 85 | 2022 | 374 |
| LDPE | Recycling | Secondary material | 548 | 141 | 3176 | 517 |
| PS | Recycling | Secondary material | 57 | 18 | 480 | 70 |
| PVC | Recycling | Secondary material | 37 | 12 | 259 | 13 |
| PET | Recycling | Disposal | 192 | 57 | 224 | 125 |
| HDPE | Recycling | Disposal | 94 | 34 | 191 | 101 |
| PP | Recycling | Disposal | 148 | 50 | 357 | 163 |
| LDPE | Recycling | Disposal | 224 | 73 | 517 | 247 |
| PS | Recycling | Disposal | 30 | 9 | 196 | 34 |
| PVC | Recycling | Disposal | 9 | 3 | 65 | 3 |
| PET | MRF | Disposal | 282 | 149 | 246 | 213 |
| HDPE | MRF | Disposal | 219 | 199 | 270 | 403 |
| PP | MRF | Disposal | 388 | 157 | 632 | 385 |
| LDPE | MRF | Disposal | 851 | 253 | 1366 | 506 |
| PS | MRF | Disposal | 235 | 56 | 364 | 90 |
| PVC | MRF | Disposal | 27 | 9 | 120 | 6 |
| PET | Mixed waste collection | Disposal | 1177 | 195 | 1229 | 140 |
| HDPE | Mixed waste collection | Disposal | 1888 | 213 | 1346 | 154 |
| PP | Mixed waste collection | Disposal | 2288 | 214 | 1353 | 154 |
| LDPE | Mixed waste collection | Disposal | 3579 | 360 | 2273 | 259 |
| PS | Mixed waste collection | Disposal | 789 | 74 | 467 | 53 |
| PVC | Mixed waste collection | Disposal | 396 | 32 | 199 | 23 |

# References

Deloitte (2017). Blueprint for plastic packaging waste: Quality sorting and recycling. Final report. Available at: <https://www2.deloitte.com/content/dam/Deloitte/my/Documents/risk/my-risk-blueprint-plastics-packaging-waste-2017.pdf> (accessed May 2020).

Dri M., Canfora P., Antonopoulos I. S., Gaudillat P., Best Environmental Management Practice for the Waste Management Sector, JRC Science for Policy Report, EUR 29136 EN, Publications Office of the European Union, Luxembourg, 2018, ISBN 978-92-79-80361-1, doi:10.2760/50247, JRC111059.

PlasticsEurope (2019). Plastics – the Facts 2018. Available at: <https://www.plasticseurope.org/application/files/6315/4510/9658/Plastics_the_facts_2018_AF_web.pdf> (accessed May 2020).

Tallentire C.W., Steubing B. (2020), The environmental benefits of improving packaging waste collection in Europe, Waste Management, 103, 426 – 436.
